# Editorial: Novel biomaterial strategies for osteogenic treatments

**DOI:** 10.3389/fbioe.2023.1137760

**Published:** 2023-01-13

**Authors:** Stephanie M. Willerth, Joshua W. Giles, Gabriella C. J. Lindberg

**Affiliations:** ^1^ Department of Mechanical Engineering, University of Victoria, Victoria, BC, Canada; ^2^ Division of Medical Sciences, University of Victoria, Victoria, BC, Canada; ^3^ Centre for Advanced Materials and Technology, University of Victoria, Victoria, BC, Canada; ^4^ School of Biomedical Engineering, University of British Columbia, Vancouver, BC, Canada; ^5^ Department of Orthopaedics, University of British Columbia, Vancouver, BC, Canada; ^6^ Department of Bioengineering, The Phil and Penny Knight Campus for Accelerating Scientific Impact, University of Oregon, Eugene, OR, United States; ^7^ Department of Orthopedic Surgery and Musculoskeletal Medicine, University of Otago, Christchurch, New Zealand

**Keywords:** biomaterials, bone, regenerative medicine, instructive scaffolds, reprogramming, protein engineered biomaterials, osteoblasts, osteogenesis

Our musculoskeletal system enables our body’s movement and function. Accordingly, diseases and disorders that inhibit these functions can have an enormous impact on the quality of life for patients suffering from them. For example, osteoarthritis results from the body’s inability to regenerate its joints after injury. Approximately 528 million people world-wide suffers from this disease, with a high prevalence (10%–14%) reported in adult populations across North America, North Africa, Middle East and Australasia by public health agencies. This disabling joint disease generates a significant socioeconomic burden on the healthcare system, with direct costs ranging between 1% and 2.5% of the gross national product, generating $65.5 billion annually in direct medical costs in the US alone (2008–2014). Likewise, the global market for bone implants is estimated to range from $40 to $70 billion, depending on the source of reporting. These implants are used to treat diseased and damaged bones and examples include metal implants as replacements for missing bone tissue or damaged hip and knee joints. Current metallic implants serve as an end-stage solution to treat predominant pain symptoms as it is costly, highly invasive and the available implants does not restore full function of the bones or joint, nor do they last a lifetime. New implant technologies, which can combine a wide range of surface modifications, signaling molecules and cells with biomaterials to instead drive the regeneration of healthy joint tissues and modulate local immune cell activation are thus highly attractive alternatives. These advanced treatment paradigms ultimately seek to fully repair and maintain the native tissue function and longevity after injection or transplantations. This Research Topic contains four research studies and one review article that have examined unique strategies for promoting osteogenesis–the generation of bone tissues.

For example, the surface properties of bone implants play a large role in their *in vivo* performance as it dictates cells’ ability to adhere, infiltrate and secrete healthy bone tissue at the implant-host tissue interface (osseointegration). Accordingly, a study from Sun et al. examined how different methods of applying hydroxyapatite coatings on 3D-printed titanium scaffolds affected their performance. They compared using plasma spray and electrochemically deposition to coat these scaffolds with hydroxyapatite and then characterized their properties both *in vitro* and *in vivo*. The use of plasma spraying resulted in a more consistent coating, with lower crystallinity and smoother surface, that was cytocompatible and promoted *in vitro* osteogenic differentiation of rat bone marrow derived stem cells. Further *in vivo* evaluation revealed an improved performance to repair a bone injury in rabbits both at early and late time-points. This study illustrates how the method of deposition affects the performance of biomaterial coatings on 3D-printed Ti-implants for bone repair by tailoring physicochemical properties of the coatings. Similarly, Wang et al. also looked a how a novel combination of surface coatings could be used to improve the performance of titanium implants both *in vitro* and *in vivo*. Here their multifunctional coating fabricated using a layer-by-layer fashion where tannic acid was deposited on hydroxyapatite followed by the application of lysozyme. This combination of materials generated both antibacterial and antioxidant effects *in vitro*, serving as a cytocompatible substrate for the culture of mouse osteoblasts. Implants containing this novel coating also demonstrated improved performance in an *in vivo* model of bone injury. Together, this pair of studies illustrate the importance of biomaterials as a tool to improve implant performance by enhancing existing materials and imbuing them with new functionality for improved osseointegration and bone healing.

Additional strategies using biomaterials to induce desirable cell-material interactions to heal bone was further examined with emerging cell reprogramming strategies by Nakai et al. The team specifically explored the use of a novel biomaterial substrate to control cell behavior through direct reprogramming, where 1 cell type (fibroblasts) was converted into the desired cell type (osteoblasts). Here, they used a novel nanomaterial called FD-NanoClip gel made up of chemically modified cholesterol-bearing pullulan. Through coating with fibronectin and culture in osteogenic media with a ALK inhibitor II, both cell attachment and cellular reprogramming of human dermal fibroblasts could be achieved. This novel biomaterial could furthermore be freeze-dried and formulated into a sheet or fibrous structures, demonstrating a difference in osteoinductive properties due to change in surface area across the two morphologies. Their results indicated that combination of protein coated biomaterials with reprogrammed cells produced high levels of calcium deposition and that architectural properties of the implant can be adjusted to guide bone healing.

As our knowledge around biomaterials and bone biology increases, the field is furthermore redirecting resources to develop biomaterials that can modulate the immune microenvironment–a complex response which plays a vital role in regulating both the speed and quality of the repair process. For example, Wang et al. designed and produced a novel recombinant protein that combined the properties of a complement immune system regulator that is unique to cartilage, the N-terminal non-collagenous domain 4 (NC4) of collagen type IX, with a heparin-binding domain to enable it to better target cells in joint tissues. They expressed the protein recombinantly in *E. coli* and verified its activity using an *in vitro* hemolytic assay to show that the modification did not affect protein function. Using a surgical-inducing osteoarthritic mouse model, they found that injections of the fusion protein (NC4-heparin) was able to localize to the joints with high retention over time. Through interactions with host cells in the joint, the delivered fusion protein further reduced inflammation and subsequently reduced loss of joint tissue. This study demonstrated how using protein engineering to generate novel biomaterials can provide novel treatments for reducing the onset of osteoarthritis by targeting complement immune system activity following joint injuries. You et al. furthermore provide a comprehensive review of how strontium can be used to functionalize biomaterials when engineering bone tissues by considering the immune response triggered. Strontium, a soft silver-white yellowish metal, is often used to dope metal implants when bone tissue engineering. This review delves into the signaling pathways and the role that this metal plays in modulating the different cells of the immune system, including macrophages and their polarization. It provides an interesting insight in considerations when using this material for tissue engineering applications. It specifically highlights the increasingly important role of understanding how the immune system interacts with bone implants to drive osteogenesis as well as the formation of blood vessels within bone tissues.

Overall, this Research Topic provides an interesting Research Topic of articles focused on novel biomaterial strategies for osteogenic treatments–ranging from protein therapeutics, surface modifications, direct reprogramming and immunoengineering. It is a timely Research Topic given the significant interest in improving health outcomes for patients suffering from diseases and disorders of the musculoskeletal system.

